# Specific GFP-binding artificial proteins (αRep): a new tool for *in vitro* to live cell applications

**DOI:** 10.1042/BSR20150080

**Published:** 2015-07-07

**Authors:** Anne Chevrel, Agathe Urvoas, Ines Li de la Sierra-Gallay, Magali Aumont-Nicaise, Sandrine Moutel, Michel Desmadril, Franck Perez, Alexis Gautreau, Herman van Tilbeurgh, Philippe Minard, Marie Valerio-Lepiniec

**Affiliations:** * Institute for Integrative Biology of the Cell (I2BC), UMR 9198, CEA, CNRS, Université Paris-Sud, Bât 430, 91405 Orsay Cedex, France; † Translational Research Department, Institut Curie, CNRS UMR 144, 26, rue d'Ulm, F_75248, Paris, France; ‡ Ecole Polytechnique CNRS UMR7654, 91128 Palaiseau Cedex, France

**Keywords:** αRep, combinatorial library, green fluorescent protein (GFP), protein design, protein interactions, repeat protein

## Abstract

A family of artificial proteins, named αRep, based on a natural family of helical repeat was previously designed. αRep members are efficiently expressed, folded and extremely stable proteins. A large αRep library was constructed creating proteins with a randomized interaction surface. In the present study, we show that the αRep library is an efficient source of tailor-made specific proteins with direct applications in biochemistry and cell biology. From this library, we selected by phage display αRep binders with nanomolar dissociation constants against the GFP. The structures of two independent αRep binders in complex with the GFP target were solved by X-ray crystallography revealing two totally different binding modes. The affinity of the selected αReps for GFP proved sufficient for practically useful applications such as pull-down experiments. αReps are disulfide free proteins and are efficiently and functionally expressed in eukaryotic cells: GFP-specific αReps are clearly sequestrated by their cognate target protein addressed to various cell compartments. These results suggest that αRep proteins with tailor-made specificity can be selected and used in living cells to track, modulate or interfere with intracellular processes.

## Abstract (precis)

Artificial proteins, named αRep, binding tightly and specifically to EGFP are described. The structures of αRep–EGFP complexes explain how αRep recognize their cognate partner. Specific αRep can be used for biochemical or live cells experiments.

## INTRODUCTION

Protein–protein interactions are essential to most biological functions in the cell. Information concerning localization, trafficking, activation states and interaction partners of proteins in living cells is crucial to understand the complexity of cellular networks. Technologies that allow modulating or preventing protein interactions offer powerful tools to investigate pathways but could also be applied to target deregulated signalling cascades in diseases [1].

Antibodies are the most commonly used scaffolds to bind protein targets. However, their high propensity to aggregation and the need for disulfide bond formation during folding are limitations for their use as intracellular tools in the reducing environment of the cytoplasm. Engineered recombinant antibody fragments of reduced size, such as single-chain variable fragment (ScFvs) are usually more efficiently produced. Some of these appear to fold *in vivo* and are compatible with intracellular applications. For example, the selection of binders from naive libraries of ScFv allowed the generation of intrabodies able to detect specific conformations of the small GTPase ras-related in brain 6 (Rab6) [2], tubulin [3] or more recently neuronal proteins such as Gephyrin and Huntingtin in living cells [4,5]. The selection process often requires an additional screen for solubility to recover soluble and stable binders from most ScFv libraries [6,7]. Single domain antibodies from camelidae (variable domain of heavy chain antibody (VHH) also called nanobodies) or shark-derived antibody fragments [8], are more soluble and efficiently expressed in heterologous systems than ScFvs; this clearly improved prospects of these molecules for a range applications including intracellular-specific VHHs [9]. For example, a GFP-binding VHH was able to capture *in vitro* and *in vivo* GFP-fusion proteins [9–11]. Irannejad et al. [12] have more recently developed VHH antibodies that detect a specific conformational state of the β2-adrenergic G-protein-coupled receptors (β2-AR GPCR), with spatiotemporal resolution in living cells. Although VHHs still need at least one essential intradomain disulfide bond, intracellular expression has been documented for some VHHs. The fraction of each VHH actually folded and functional in reducing conditions presumably varies with the stability of each molecule. Additionally, efficient processes to obtain VHH binders currently rely on camelidae immunization, followed by phage display selections from ‘immune’ libraries and screening of the best candidates. Therefore, until efficient naive library become available, VHH technology is not optimal to generate binders when a clear control on the target molecular state is required.

Non-antibody scaffolds offer an alternative and very attractive approach for the creation of protein recognition tools. Synthetic large protein libraries with highly randomized binding surfaces are derived from a stable protein scaffold, which is diversified at specific positions. The few variants of a library binding tightly and specifically to any specific given target can be selected out by phage or cell display methods. Scaffold candidates should be soluble, stable and disulfide-free to prevent inefficient folding in a reducing environment. One important example of a non-antibody scaffold is provided by the tenth type III fibronectin domain (named monobodies) [13]. Interesting intracellular applications were reported for monobodies; for example, they were used to detect specific conformational changes of the oestrogen receptor in a living cell [14]. They were also applied as highly selective inhibitors directed against the Src homology 2 (SH2) domains of SH2 domain-containing phosphatase 2 (SHP2) phosphatase in order to dissect the signalization cascade of the break point cluster region-gène abelson (BCR-ABL) oncogene protein by specifically interfering with targeted protein domains [15] or as fusion with GFP to track PSD95 and Gephyrin in neuron in real time [16].

Repeat proteins are an emerging class of alternative scaffolds for the creation of protein binders to specific intracellular probes. These types of proteins result from the repetition of a simple motif typically long from 20 to 40 amino acids and fold in solenoid-like architecture. In the folded proteins the juxtaposition of each motif generates an extended surface very well adapted to macromolecule recognition. Several types of repeats as leucine rich-repeat (LRR) [17,18] tetratricopeptide (TPR), armadillo, HEAT and ankyrin repeats have been used as molecular template to develop large libraries of binding scaffolds [18,19]. Intracellular applications of engineered repeat proteins were first successfully achieved with designed ankyrin repeat proteins (DARPins), [20] and TPR [21]. Recent applications clearly confirm the potential of engineered repeat proteins as tailor made intracellular recognition units [22–24].

We here present a new type of repeat proteins, the αRep proteins, as a tool for specific molecular recognition of protein targets inside living cells. Apart from monobodies or DARPins, only few examples of non-antibodies-derived artificial proteins selected from libraries have been described so far for their abilities to bind/track/modulate intracellular targets. Synthetic libraries can offer versatile sources of these intracellular binders and the development of different scaffolds can enlarge the choice for the right probe to any cellular application. The construction of a library of artificial repeat proteins called αReps was previously described [25]. Sequence alignment of a subfamily of natural thermostable HEAT repeat proteins helped to design a consensus repeat sequence coding for a motif of 31 residues containing five highly variable positions. Polymerization of degenerated microgenes coding for a motif, randomized at the variable positions in between specific N-terminal and C-terminal sequence, generated a highly diverse library of 1.7×10^9^ independent clones. Proteins from this library were well expressed, soluble and stable. They vary in sequence at variable positions and in length depending of the number of inserted repeats [25]. Using an optimized phage display library, specific αReps could be selected, with micromolar to low nanomolar dissociation constants for various protein targets [26]. The present paper is focused on the selection and detailed characterization of αReps tailored to interact with fluorescent proteins.

The discovery and development of fluorescent proteins have revolutionized the studies of proteins in living cells and organisms, as they are genetically encoded fluorescent tags. The GFP is now widely used by biologists. *S*pecific GFP binders could therefore be used for the purification of GFP fusion proteins as well as to track GFP-fusion proteins in a cellular context [27]. Other interesting applications of GFP binders have been described such as induced protein degradation [28] or control of gene expression [29]. Very recently, a set of DARPins binding specifically to fluorescent proteins has been described [30]. These DARPin-based GFP binders are functional in living cells and can be used in cell and developmental biology for protein tracking and protein interference experiments.

Clearly, well-characterized artificial proteins binding to fluorescent proteins are potentially useful tools for a large community. A first αRep protein binding GFP has been reported [26]. We present here two additional αRep proteins able to recognize GFP with high specificity and affinity. The X-ray structures of both αReps in complex with GFP surprisingly showed that they adopt totally different binding modes. The ability of αReps to isolate their target in a crude cell lysate was confirmed by a pull-down experiment. Finally, we were able to express the αReps in mammalian cells and their localization was modified by the interaction with the GFP protein target addressed to different cellular compartments (mitochondria, Golgi apparatus and nucleus).

## MATERIAL AND METHODS

### Phage display selection against biotinylated EGFP

αRep library 2.1 was used to perform phage display selection. The selection methods were as previously described [26]. Briefly, *in vivo* biotinylated EGFP was linked on streptavidin-coated micro-titre ELISA plate. To prevent the selection of streptavidin-binding clones, phages from the library were pre-incubated in wells coated with streptavidin (1–2×10^10^ phages/well) and then transferred in the selection plate for 1 h at 20°C. After several washes with Tris-buffered saline and Tween 20 (TBST-20 mM Tris/HCl, pH 8.0, 150 mM NaCl, 0.1% Tween-20) bound phages were specifically eluted by releasing immobilized GFP with TEV protease (10 μg of ml^−1^) for 3 h at 25°C.

### Screenings of αReps for target binding

After three rounds of selection, specific clones were identified by phage-ELISA [26] and further confirmed using a functional CoFi-blot analysis [31,32]. This test detects the presence of biotinylated target retained on phage-free αRep directly expressed from bacterial colonies. Biotinylated target bound on positive clones were detected by fluorescence imaging using streptavidin-Alexa-680.

The sequence of 11 clones presenting strong binding signals for the target revealed four different αRep protein sequences among which three were further characterized.

### Proteins expression and purification

αRep variants and fluorescent proteins (FPs) genes were sub-cloned in pQE81L vectors (Qiagen). Expression and purification of αRep and GFP proteins were performed as described [25]. The plasmid coding for each protein was transformed into the expression strain (BL21-Gold DE3 Agilent). Cells were grown at 37°C in 2YT containing 100 μg·l^−1^ ampicillin up to an absorbance of 0.6 at 600 nm. Protein expression was induced by addition of 1 mM IPTG and the cells were further incubated for the four αReps at 30°C. The cells were then harvested, suspended in TBS, submitted to three freezing/thawing cycles, treated with benzonase for 30 min and sonicated.

Biotinylated αRep used for pull-down experiments were produced using the Avitag system. αRep coding sequences were sub-cloned in a modified pQE81L vector in which the biotinylation sequence (GLNDIFAQKIEWHE) was added in phase in between the His-tag and the αRep cloning sites.

The His-tagged proteins were all purified from crude supernatant using nickel-affinity chromatography (nickel-nitrilotriacetic acid (Ni-NTA) agarose, Qiagen) followed by size-exclusion chromatography (SEC; Hiload 16/60 SuperdexTM 75 GE Healthcare) in PBS or HEPES buffer. For each protein, purity of the final sample was controlled by SDS/PAGE with an overloaded gel showing one well-resolved band with no visible contamination. For all the following experiments the proteins were quantified by UV spectrophometry (280 nm) and expressed in monomer concentration.

### Analytical size-exclusion chromatography

Analytical SEC was done with an ÄKTA Purifier (GE Healthcare) system using a Superdex™ 75 10/300 column (flow-rate 0.8 ml·min^–1^) equilibrated in PBS. For all the purified proteins analysed, 100 μl of protein sample (1–15 nmol depending on experiments) were injected on to the column. For each elution profile, *A*_280_ nm was normalized relatively to its maximum.

### Isothermal titration calorimetry

The binding parameters were monitored with an isothermal titration calorimetry (ITC) 200 microcalorimeter (MicroCal). For the titration of target protein, 2 μl aliquots of the titrant (varying from 350 μM to 364 μM, depending on the experiment) were injected from a computer-controlled 40-μl microsyringe at intervals of 180 s into the solution of target (varying from 30 to 35 μM, depending on the experiment; cell volume 0.24 ml) dissolved in the same standard buffer (PBS) while stirring at 1000 rpm. The heat of dilution of the binder was determined from the peaks measured after full saturation of target by the binder. The data were integrated to generate curves in which the areas under the injection peaks were plotted against the ratio of injected sample to cell content. Analysis of the data was performed using the MicroCal Origin software provided by the manufacturer according to the one-binding-site model. Δ*H*°, the standard change in enthalpy and Δ*G*° the standard change in Gibbs free energy were calculated by integration of heat capacity variation from the titration curve and associated equilibrium constant. Δ*S*° is the standard change in entropy upon binding was calculated from determined equilibrium parameters using the equation: −*RT*Ln(*K*_A_)=Δ*G*°=Δ*H*° − *T*Δ*S*°, where *R* is the universal gas constant (1.9872 cal·mol^−1^·K^−1^), *T* is the temperature in Kelvin, *K*_A_ is the association constant. The binding constant of each interaction is expressed as 1/*K*_A_=*K*_D_ (in mol·l^−1^) for more clarity.

### Surface plasmon resonance

Surface plasmon resonance was measured using a Proteon™ XPR36 instrument (Bio-Rad). All measurements were performed in 50 mM phosphate buffer, pH 7, 150 mM NaCl and 0.005% Tween 20 at a flow rate of 100 μl·min^−1^. ProteOn GLC sensor chip (Bio-Rad) were used to immobilized αRep proteins (bGFP-A, bGFP-C, bGFP-D and αRep A3) in parallel on one of the six channels chip following the amine-coupling protocol. For the determination of kinetics data, purified FPs (EGFP, ECFP, EYFP and mCherry) were injected each at six different concentrations in parallel (0; 1.1; 3.3; 10; 30 and 90 nM) during 200 s and dissociation signals were acquired during 600 s. The signal of the uncoated reference channel and interspots were always subtracted from the sensorgrams. The kinetic data were analysed with the Proteon Manager software fitted by Langmuir analysis for the five protein concentrations.

### Crystallization, structure determination and refinement

All crystallization experiments were carried out at 293 K using the vapour diffusion method. Initial crystallization screening was done at three different protein concentrations (15, 10 and 5 mg·ml^−1^) using commercially available kits (Qiagen Classic, MB Class I, PEG II, JCSG+). The two complexes behaved rather differently during the crystallization process. The bGFP-A–EGFP complex crystallized under many different crystallization conditions, whereas for the bGFP-C–EGFP complex only one hit was obtained. Optimization of the initial hits led to the following crystallization conditions: complex bGFP-A–EGFP (0.05M MgAc, 0.1M NaAc, 5%–15% PEG 8K) and complex bGFP-C–EGFP (50 mM Tricine, pH 6.9, 25% PEG4K).

Crystals were flash-frozen in liquid nitrogen by two soaking steps using mother liquor supplemented with 15% and 30% glycerol as cryoprotectant. Diffraction data were collected at 100 K on beamline PROXIMA 1 at the SOLEIL synchrotron using a PILATUS detector. The images were integrated with the XDS program [33] and processed using the CCP4 program suite [34]. For the resolution of the structure, molecular replacement phases were obtained with Phaser, implemented in the CCP4 program suite [35] using the following search models: the structure of EGFP from *Aequorea victoria*, PDB ID 1JBZ [36] and a six-helix motif from the αRep4 structure, PDB ID 3LTJ [23]. The experimental map was improved by solvent modification using the program DM [37]. The initial models were completed and adjusted with the program COOT [38]. Refinement was performed using REFMAC [39]. The crystal structure of the bGFP-A–EGFP (complex 1) at 2 Å (1 Å=0.1 nm) resolution was refined to *R* and *R*_free_ crystallographic factors of 20.5% and 26.8% respectively (Table 1). The crystal structure of the bGFP-C–EGFP (complex 2) at 3.4 Å resolution was refined to *R* and *R*_free_ crystallographic factors of 20.9% and 29.2% respectively (Table 1). Atomic co-ordinates and structure factor were deposited in the Protein Data Bank under accession codes 4XL5 for the complex 1 (bGFP-A–EGFP) and 4XVP for the complex 2 (bGFP-C–EGFP) respectively.

**Table 1:** Presentation of the three αReps selected against EGFP: bGFP-A, bGFP-C and bGFP-D For each protein the number of inserted internal repeats in between N-cap and C-cap and the nature of the residues found at randomized positions are indicated. Eighteen, 19, 22, 23, 26, 30 refers to the position of the residues inside each repeat. Only residues E, K and Q are allowed at position 30. For each αRep kinetic binding constants, *k*_on_, *k*_off_ and dissociation constant *K*_D_ for the FP variants (EGFP, EYFP and ECFP) were measured by SPR (Proteon Biorad). αReps were immobilized to the chip surface by amine coupling and different concentrations of each variant were injected (90, 30, 10, 3.3, 1.1 and 0 nM). Dissociation constants of the three αReps for the EGFP proteins were also measured by ITC (in the syringe: bGFP-A: 350 μM; bGFP-C: 364 μM; bGFP-D in the cell: 30 μM and EGFP in the cell: 35 μM or in the syringe: 298 μM).

	Motifs number		Variable sequence positions									*K*_D_ (nM)	
αRep		Motif	18	19	22	23	26	30	Variant	*k*_on_ 10^4^ M^−1^·s^−1^	*k*_off_ 10^−4^ s^−1^	SPR	ITC
bGFP-A	6	Ncap	P	P	V	Y	F	K					15±4
		1	A	S	Y	A	T	Q					
		2	G	Y	T	A	E	Q	EGFP	15±1	2.1±0.1	1.4±0.1	
		3	P	W	L	T	R	E	EYFP	12±1	16±1	14±1	
		4	P	W	L	T	R	Q	ECFP	11±1	6.9±0.1	6.5±0.1	
		5	A	S	K	A	V	Q					
		6	E	Y	Q	R	S	K					
bGFP-C	3	Ncap	M	R	Y	N	T	K	EGFP	6.2±0.1	2.6±0.1	4.2±0.1	19±12
		1	G	Y	L	E	E	E	EYFP	11±1	6.3±0.1	5.5±0.1	
		2	P	D	S	E	L	K	ECFP	5.8±0.1	14±1	25±1	
		3	R	Y	M	A	W	K					
bGFP-D	4	Ncap	M	P	Y	D	D	K					—
		1	P	N	A	S	D	K	EGFP	2.9±0.1	4.2±0.1	14±1	
		2	G	Y	F	S	L	K	EYFP	7.9±0.1	1.7±1.1	2.1±0.1	
		3	S	R	W	S	Y	Q	ECFP	7.6±0.1	9.0±0.1	11±1	
		4	W	Q	K	A	V	K					

### Pull-down experiment

Human embryonic kidney cells (HEK) (10×10^6^), transfected with the plasmid pEGFP-N1 (Clontech), were harvested 48 αRep after transfection and lysed in 1% Triton X100, 150 mM NaCl, 50 mM Tris/HCl, pH 7.4, 1 mM EDTA during 30 min at 4°C. Cellular lysate was ultra-centrifuged at 90000 ***g*** for 30 min. The supernatant, containing EGFP proteins, was divided in five aliquots, each incubated with either 300 μg of biotinylated αRep (bGFPs and A3) or dilution buffer (150 mM NaCl, 50 mM Tris/HCl, pH 7.4), all adjusted to a final volume of 1.5 ml, during 3 h at 4°C. Supernatant (7.5 μl) was diluted in SDS containing buffer to be analysed (referred as input). Streptavidin-Agarose beads (40 μl; Thermo Scientific), previously equilibrated in wash buffer (150 mM NaCl, 50 mM Tris/HCl, pH 7.4, 0.1 mM EDTA), were added in each mix and incubated 1 h at 4°C. After a centrifugation step (2 min, 3000 ***g***, 4°C), supernatants were isolated and 7.5 μl was used for SDS page (referred as depleted lysate). Beads were quickly washed four times (1000 ***g***, 15 s, in 1 ml of 0.16% Triton X100, 150 mM NaCl, 50 mM Tris/HCl, pH 7.4, 0.16 mM EDTA). Beads were suspended in 4× SDS-containing buffer.

SDS/PAGE (12% gel) were used for samples migration. GFP proteins on western blot (WB) nitrocellulose membrane were revealed using anti-GFP antibody (Cell Signaling) and anti-rabbit IgG–HRP immuno-conjugate, observed by addition of Clarity™ western ECL substrate (Bio-Rad).

### Eukaryotic cell expression

αReps sequences were sub-cloned in a modified pmCherry-N1 vector (Clontech) in which Flag-tag sequence was added in N-terminal of the multiple cloning site (MCS). Plasmids contained EGFP genes were previously described (pEGFP-Rab6 [2], pEGFP-mito [40], pNLS-EGFP [41]). HEK cells were grown in Dulbecco's Modified Eagle medium (DMEM)-F12 medium supplemented with 10% FBS and HELA cells were grown in DMEM, high glucose, GlutaMAX (Lifetechnologies) containing 10% heat-inactivated fetal calf serum (FCS), 1 mM sodium pyruvate. Transfections were realized using Lipofectamine LTX (Invitrogen) following the commercial protocol for HEK cells and thanks to calcium phosphate for HELA cells. For microscopy, 10^5^ HEK cells were directly transfected in micro slide eight-well (Ibidi) with αRep plasmid or αRep and GFP plasmid and examined after 1 or 2 days.

### Microscopy

Live cells expressing fluorescent proteins were analysed using Axio Observer microscope (Zeiss, 40× objective) or confocal spinning disk Yokogawa CXU-X1 A1 microscope (60× objective) at 37°C, with CO_2_.

## RESULTS

### Selection from the library of GFP-binding αReps

The αRep scaffold, the library construction and the phage display selection procedure against the biotinylated target EGFP were previously described [26]. Briefly, three rounds of selection were performed using the αRep 2.1 library against biotinylated EGFP bound to streptavidin in an ELISA Plate. The GFP-binding clones were identified using successively a phage-ELISA screen and a functional colony filtration blot. In the phage-ELISA experiment, bacteriophages produced from individual αRep clones are incubated in presence of the immobilized target and revealed using an anti-phage antibody. In the functional colony filtration blot, the proteins from soluble cytoplasmic fractions of isolated clones are adsorbed on a nitrocellulose membrane and incubated with the biotinylated target; the bound target protein is revealed using fluorescent streptavidin. Clones showing affinity for the target were sequenced and redundancy could be observed in those sequences obtained after the third round of selection (four unique sequences out of 24 clones). Three binders of interest were further characterized. One of the sequences, bGFP-A, was described [26] and the two others will be referred as bGFP-C and bGFP-D. A fourth sequence (bGFP-B) displayed strong binding signals but this purified protein appeared by SEC to associate in a range of oligomeric forms and for this reason was not further investigated. The three selected binders differ both in the number of inserted repeats they contain (respectively 6, 3 and 4 motifs) and in residues that are found at the randomized positions (Table 1).

The isolated αRep genes were sub-cloned in expression vector (pQE81L), produced in *Escherichia coli* and purified using immobilized metal affinity chromatography (IMAC) followed by SEC. They are all very well expressed (from 50 to 100 mg·l^−1^), soluble and stable as already observed for proteins from the αRep libraries.

### *In vitro* characterization of EGFP–αRep complexes

#### Analytical size exclusion chromatography

Analytical SEC was used to determine the quaternary structure of the αRep proteins and their complexes with GFP in solution. Previous SEC results showed that bGFP-A protein eluted as a dimer, although it forms a 1:1 complex with EGFP [26]. A solution containing bGFP-C or bGFP-D with GFP was injected on an analytical SEC column (Figure 1A; Supplementary Figure S1A). The proteins bGFP-C, bGFP-D and EGFP are eluted respectively at 11.2 ml, 11.5 ml and 11.3 ml. For each mixture, a new peak was observed at a lower volume (10.5 ml for bGFP-C and 8.9 ml for bGFP-D) compared with those of the target or binder protein alone. This is consistent with the formation of a GFP–αRep complex.

**Figure 1:**
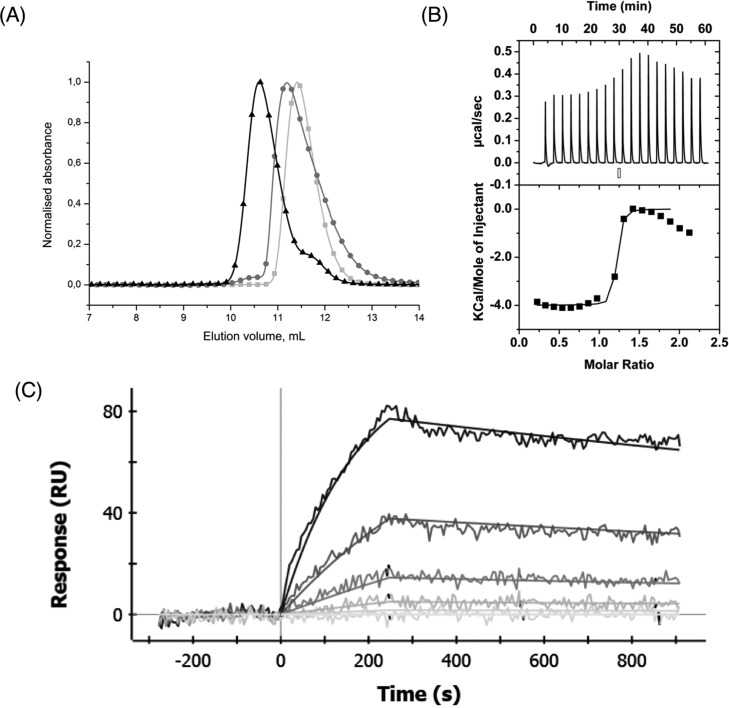
Biophysical characterization of the bGFP-C–EGFP complex (**A**) SEC (Superdex 75 10/300 GL, GE Healthcare) of the selected bGFP-C and EGFP. (▲) SEC Elution profile of a mixture of EGFP (4 nmol) and binder bGFP-C (4 nmol); (●): elution profile of the bGFP-C alone (2.2 nmol); (ü): elution profile of the EGFP alone (6.7 nmol). (**B**) ITC calorimetric titration of EGFP (35 μM) with bGFP-C (364 μM). (**C**) Affinity determination of selected bGFP-C using SPR. In this example, different concentrations of EGFP respectively 90, 30, 10, 3.3, 1.1 and 0 nM, were applied for 200 s, followed by washing buffer flow during 600 s, to a flow cell where bGFP-C was immobilized by amine coupling. The sensorgrams were corrected for non-specific binding by subtraction of the signal obtained in channel without EGFP (0 nM) injected. Proteon Manager software was used to fit the curves by Langmuir analysis and determine the *k*_on_ and *k*_off_ rates and then deduce the *K*_D_.

In summary, SEC indicates that each of the three αReps forms a complex with GFP stable enough to be isolated.

#### Binding affinity determination

ITC experiments were performed to determine the affinity of the binders for the EGFP target. The dissociation constants for bGFP-A–EGFP and bGFP-C–EGFP complexes were found to be in the nanomolar range, with *K*_D_ values of respectively 15±4 nM [26] and 19±12 nM (Table 1; Figure 1B). For bGFP-A and bGFP-C, the stoichiometry values (*n*) of 1.1 and 1.2 respectively indicated the formation of 1:1 complexes. However for bGFP-D, no apparent binding signal could be measured. The lack of ITC signal of the bGFP-D for the EGFP was unexpected given that a stable interaction is unambiguously observed by various other techniques (ELISA, CoFi Blot, gel filtration). The lack of ITC signal for the interaction between bGFP-D and EGFP suggested that the enthalpic contribution is counterbalanced by an opposite sign entropic contribution, cancelling the resulting measured signal.

In order to complete the ITC results, surface plasmon resonance (SPR) experiments were carried out to determine the binding constants of each αRep for the EGFP target. For the complex bGFP-D–EGFP, the SPR-measured equilibrium dissociation constant (*K*_D_) was 14±41 nM. The *k*_on_ values were found in the order of 10^4^ M^−1^·s^−1^ showing a rapid binding on the target and a slow dissociation rate (order of magnitude of *k*_off_: 10^−4^ s^−1^). The nanomolar range of the *K*_D_ values for the complex formation of bGFP-A (1.4±0.1 nM) and bGFP-C (4.2±0.1 nM) was confirmed. *K*_D_ values obtained by SPR were smaller than those measured by ITC. This origin of differences between ITC and SPR *K*_D_ values are not fully understood but similar discrepancies have already been observed [42]. In our case, part of the differences may be related to the fact that, due to high affinities, ITC curves contained only few points within the transition part of the saturation curve and which lead to an increased error on *K*_D_ values.

#### Binding specificity

In order to analyse the specificity of each binder, the interactions with various FPs were measured by SPR. EGFP, ECFP, EYFP and the mCherry protein were purified by affinity chromatography (IMAC) followed by SEC. The FP variants of the EGFP, ECFP and EYFP, differ only in residues near the chromophore, which is buried inside the β-barrel of the protein and in a few surface residues. As shown in Table 1, only slight binding-affinity differences between the EGFP, ECFP and EYFP and the GFP binders were observed. This result can be rationalized with the known structures of αRep–EGFP complexes (see below) that indicate that the surface residues that differ between EYFP and ECFP are not located in the αRep-binding surface. The binders bGFP-A, bGFP-C and bGFP-D could thus be used as generic binders for these closely related FP. Conversely, bGFP binders had no affinity for the mCherry protein, which has the same β-barrel fold but displays more differences in surface residues.

In order to understand how the selected αReps of different length bind their common target with comparable affinities, the structures of two of the studied complexes have been investigated by X-ray crystallography.

### X-ray structures of two complexes

#### Structure determination of the EGFP–bGFP complexes

bGFP-A–EGFP (complex 1) crystallized in the monoclinic space-group P2_1_ and crystals diffracted at 2 Å resolution (Table 2). The asymmetric unit contains one copy of the hetero-dimer. Clear electron density was observed for residues 3–231 of the EGFP and 15–259 of the bGFP-A αRep. bGFP-A consists of 16 α-helices, which superposed well with the last 12 helices of a previously described [25] αRep-4 structure (RMSD value of 0.367 Å for 168 atoms superimposed; Figure 2A).

**Table 2:** X-Ray data collection and refinement statictics

PDBID	bGFP-A–EGFP 4XL5	bGFP-C–EGFP 4XVP
Data collection		
Beamline	PX1 (SOLEIL)	PX1 (SOLEIL)
Wavelength (Å)	0.97857	0.97918
Space group	P21	C2
Unit cell dimensions		
a, b, c (Å)	72.28, 45.98, 74.89	105.52, 71.55, 179.20
α, β, γ (°)	90.00, 90.76, 90.00	90.00, 100.72, 90.00
Resolution (Å)*	39.2-2.0 (2.1-2.0)	46.7-3.4 (3.6-3.4)
Completeness (%)	98.5 (98.1)	99.7 (80.1)
Multiplicity	3.0 (3.0)	3.5 (2.3)
I/σ (I)	14.3 (2.0)	10.9 (2.0)
*R*_merge_^†^	0.05 (0.77)	0.10 (0.54)
Number of reflections	99062 (15572)	61834 (6295)
Number of unique reflections	33143 (5270)	19164 (2826)
Wilson B factor (Å)	46.155	48.647
**Refinement**		
Resolution (Å)*	39.2-2.0 (2.1-2.0)	46.7-3.4 (3.5-3.4)
R-work	0.205 (0.385)	0.209 (0.397)
R-free	0.268 (0.377)	0.292 (0.43)
Number of non-H atoms	3790	9144
EGFP	1818	5472
bGFP	1869	3672
Water	103	-
RMSD bonds (Å)	0.014	0.010
RMSD angles (°)	1.7	1.4
Ramachandran favoured (%)	99.57	98.94
Ramachandran outliers (%)	0.43	1.06
Average B-factor (Å^2^)	49.12	118.36
EGFP (Å^2^)	48.33	120.1
bGFP (Å^2^)	50.11	115.78
Water (Å^2^)	45.37	–

*Statistics for highest resolution shell are shown in parentheses.

†*R*_merge_=ΣhΣi|Ihi - <Ih>|/ΣhΣiIhi, were Ihi is the *i*th observation of the reflection h, whereas <Ih> is the mean intensity of reflection h.

**Figure 2:**
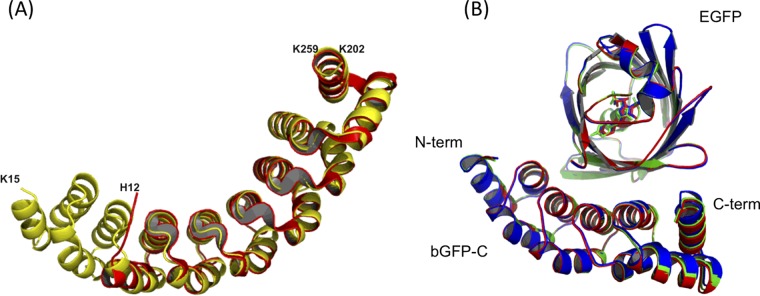
structures of GFP-binding αReps (**A**) Superposition of the 16 α-helices of bGFP-A (yellow) with the 12 α-helices of αRep-4 (red, PDB ID 3LTJ; [53]), RMSD=0.367 Å. (**B**) Superposition of the three hetero-dimers (A:D, B:E and C:F) of the complex 2 (bGFP-C–EGFP) in green, blue and red, from the same asymmetrical unit (RMSD=0.167 Å for 330 residues and RMSD=0.140 Å for 318 residues.

bGFP-C–EGFP (complex 2) crystallized in the monoclinic C121 space group and crystals diffracted at 3.4 Å resolution (Table 2). Complex 2 contains three hetero-dimers in the asymmetric unit. The refined structure consists of residues 2–231 for EGFP (chains A, B and C) and residues 9–166 for bGFP-C (chains D, E and F). The three hetero-dimers are structurally identical with a RMSD of 0.167 Å and 0.140 Å for superimposed Cα-positions (Figure 2B).

bGFP-A and bGFP-C adopt the canonical αRep fold consisting of 6 and 3 α-helical internal repeats respectively and well-defined C- and N-caps. EGFP undergoes only very limited structural changes upon binding the bGFPs.

The interface areas (as calculated by PISA [43]) for complex 1 are 934 Å^2^ (bGFP-A–EGFP) and for complex 2, 1309 Å^2^ (bGFP-C–EGFP), which is in the range of standard protein/protein interfaces [44,45]. Although the size of bGFP-C is two times smaller than for bGFP-A, its interaction surface with EGFP is larger by 375 Å^2^.

Surprisingly, the two binders interact very differently with their EGFP target in their respective complexes. Although their N-caps bind to the same region on EGFP, the interaction modes and the relative orientations of the binders are radically different. In complex 1 the helices of bGFP-A are oriented perpendicularly to the barrel axis of EGFP, whereas, for complex 2, the helices of bGFP-C are parallel with the barrel axis (Figure 3). The majority of the direct protein contacts between bGFP-A and EGFP surface are located at the N-cap helices of the repeat protein. The curvature of bGFP-A creates a cavity between the EGFP barrel surface and its central repeats (R5 and R6) that is filled with water molecules that mediate indirect protein interactions. The direct interactions between bGFP-A and EGFP are mainly hydrophobic and mediated by randomized residues on the concave surface of the proteins (Figures 4A and 4B). However, only 14 out of the 42 randomized residues are involved in the interaction. Those positions that interact are situated mainly on the N-terminal part of each repeat (positions 18 and 19 on the repeat) since the C terminal parts of the repeats are not in contact with the EGFP surface. For example, positions 19 of repeat 1, 2, 3, 4 and 6 generate 38 out of the 101 hydrogen bonds of the interface. The only three hydrogen bonds observed in the structure are located in the N- and C-terminal of the binder (Tyr^33^ of the N-cap, Asp^58^ and Arg^219^ in the R1 and R6 repeat respectively). Hydrogen bonds « via » waters molecules are also observed in the repeats R1, R2 and R3: S^60^-HOH^44^-K^41^ (repeat 1); Y^91^HOH^27^-S^208^ (repeat 2); W^122^-HOH^26^-L^207^ (repeat 3). Except in the N-cap, the variable positions 26 and 30 are not involved in the interaction. Unexpectedly, a non-randomized residue (Asp^58^) also participates in the interaction, forming a hydrogen bond with EGFP Tyr^39^. The N-cap and the last internal repeat (R6) play an important role in the interface, generating two of three directs hydrogen bond observed (Tyr^33^ and Arg^219^). Overall, the strong interaction measured between bGFP-A and EGFP almost entirely originates from randomized side chains but not all randomized side chains interact. The αRep–EGFP interaction thus does not exploit the whole potential of the αRep surface. The selected αRep bind EGFP efficiently, although, due to the partial sampling of the sequence space, each binder is unlikely to display the ′optimal′ side chain combination for its binding surface. Further optimization of such large surfaces could be conducted using affinity maturation methods.

**Figure 3:**
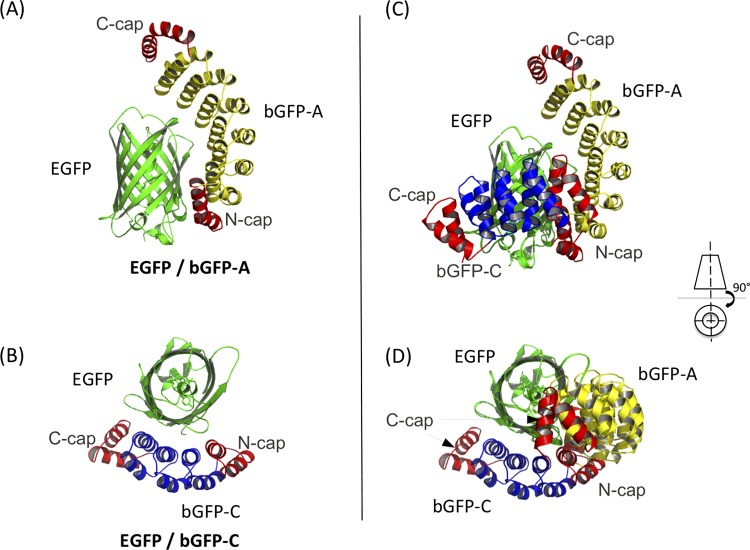
Details of the complexes structures: capping repeats are represented in red, EGFP in green, bGFP-A in yellow and bGFP-C in blue (**A**) EGFP–bGFP-A complex, side view; (**B**) EGFP–bGFP-C complex, top view; superposition of both complexes structures: (**C**) side view and (**D**) top view, N-cap motif interact with the same GFP region

**Figure 4:**
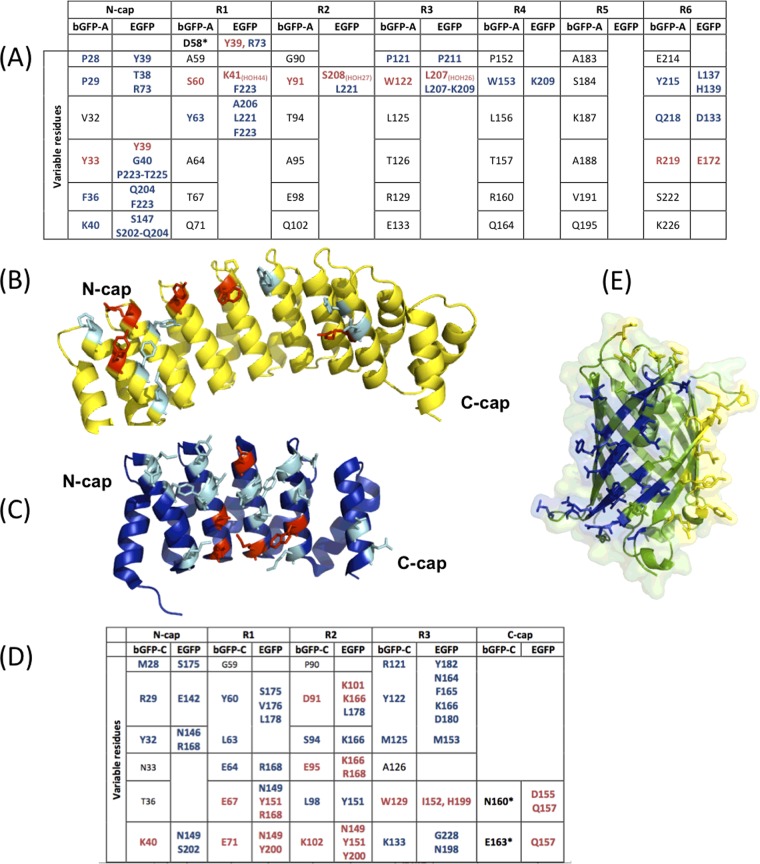
Analysis of the EGFP–bGFP interactions Hydrophobic contacts are coloured in light blue in the tables and in cyan in the structures, hydrogen bonds are in red and salt bridges in orange. *conserved residues of αReps involved in specific interaction with the target. (**A**) Table presenting variable residues of bGFP-A for each repeat [N-Cap and repeats 1–6 (R1–R6)]; coloured residues contact with EGFP, residues in black are not involved in the interaction. (**B**) Cartoon representation of the bGFP-A structure in yellow, residues involved in the interaction are represented as sticks and coloured according to the nature of the interaction with EGFP residues. (**C**) Cartoon representation of the bGFP-C structure in dark blue, residues involved in the interaction are represented as stick and coloured according to the nature of the interaction. (**D**) Table presenting variable residues of the bGFP-C arranged according to their position in each repeat [N-Cap, repeats 1–3 (R1–R3) and C-cap], coloured residues are involved in the main contact with EGFP residues, residues in black are not involved in the interaction. (**E**) EGFP structure in green, residues involved in the interaction with αReps are represented as sticks: in yellow, residues interacting with bGFP-A and in blue, residues interacting with bGFP-C. No residue of the GFP involved in interaction is found in both complexes.

The interaction surface between bGFP-C and EGFP is quite different from that of complex 1: bGFP-C has less repeats than bGFP-A and almost all of its diversified residues are involved in the interaction: 23 out of 24 randomized residues are located in the interface and 21 out of 24 randomized residues are involving in contacts with EGFP from chains D, E, F respectively. Unlike previously observed for the four other αRep structure complexes already solved, the not randomized C-cap module is also involved in the interaction. The details of all the interactions are presented in Figure 4(A).

#### Energetic profiles of the interactions

Although the binding affinities of bGFP-A and bGFP-C for EGFP are very similar, the crystal structures of the complexes showed they bind very differently to the EGFP protein. Despite their different binding modes, the two binders mainly interact with EGFP via the randomized surface residues situated on their concave surfaces. A more detailed analysis of the interfaces between the αRep proteins and EGFP revealed that different types of interactions stabilize the complexes. The thermodynamic parameters of binding obtained by ITC are presented in Figure 5, showing that both binders have compensating differences in their enthalpic and entropic contributions. bGFP-A, has an unfavourable ∆*H* [5.58±0.06 kcal·mol^−1^ (1 cal≡4.184 J)] and a favourable negative −*T*∆*S* (−16.33 kcal·mol^−1^) contribution. The favourable entropic energy (−*T*∆*S*) suggests that binding is mainly driven by hydrophobic interactions. This result is corroborated by the structural analysis showing that complex formation involves 10 hydrophobic residues located between the N-cap and the fourth repeat (Figure 4A). The thermodynamic analysis of the bGFP-C binding to EGFP presented in Figure 5, shows both favourable enthalpic (−3.99±0.07 kcal·mol^−1^) and entropic contributions (−6.37 kcal·mol^−1^). The favourable enthalpic contribution reflects the important involvement of hydrogen bonds in this interaction, as corroborated by the structure: the bGFP-C–EGFP complex involves 165, 154 and 156 contacts (Figure 4D) with 9, 9 and 8 direct H-bonds for A:D, B:E and C:F heterodimers respectively. On the other hand, the favourable entropic contribution is probably due to the burial of the hydrophobic groups and release of water upon binding in the two hydrophobic patches present at the interface (bGFP-C Tyr^60^; Leu^63^ with EGFP Ser^175^; Val^176^; Leu^178^ and bGFP-C Arg^121^; Tyr^122^; Met^125^ with EGFP Tyr^182^; Phe^165^; Asn^164^; Met^153^).

**Figure 5:**
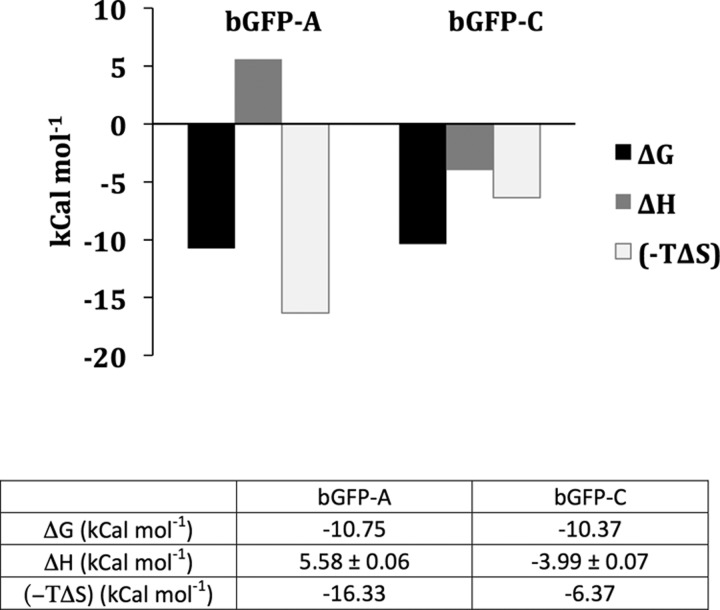
Energy balance of interactions of both complexes bGFP-A–EGFP and bGFP-C–EGFP Plot of entropic and enthalpic contributions resulting of the interaction of each complex, determined by ITC. Δ*H*, enthalpic contribution; −*T*Δ*S*, entropic contribution.

### αReps as tools for biochemical and functional studies in living cells

In order to determine whether artificial αRep proteins could be used in living cells, we characterized the αRep–EGFP interactions in a cellular context.

#### αReps can pull down their target from a cellular extract

According to the high affinity measured *in vitro* for the interaction, αRep proteins should be able to selectively bind their target within a complex mixture such as crude cell extract. To confirm this hypothesis, we set up a pull-down experiment. αReps can easily be expressed and biotinylated in *E. coli* by the addition of an Avi-tag sequence to the N-terminus. The individual previously purified biotinylated bGFPs and a non-relevant αRep used a control, were incubated with a cell lysate of HEK 293 cells expressing EGFP. αReps were captured on streptavidin-agarose beads and aliquots of the remaining unbound supernatants were kept for analysis (depleted lysate). Following washing steps, the bound αRep complexes were denatured with SDS and analysed by western blot using an anti-GFP antibody (Figure 6). No EGFP was detected in the depleted lysate incubated with each of the bGFPs whereas, in control experiments, EGFP remains in the lysate in absence of αRep or in presence of non-relevant αRep. These results show that bGFPs are able to capture EGFP from a complex mixture. A clear band on the western blot for EGFP was detected in the eluted fractions from beads that bound bGFPs, but not from the controls. The αReps bGFPs are clearly able to retain specifically their target after repeated washes and can be used as tools to isolate their target from a complex cell extract.

**Figure 6:**
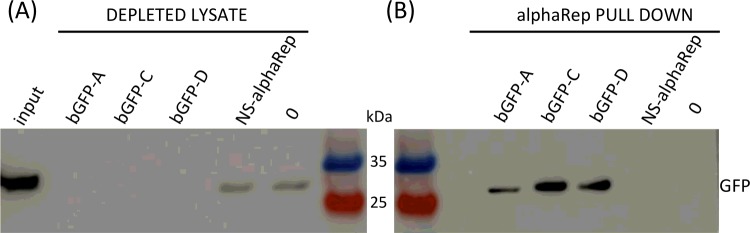
Affinity purification of EGFP protein by specific αRep proteins Biotinylated αRep proteins were attached on streptavidin-agarose beads: bGFP-A, bGFP-C, bGFP-D, NS-αRep (non-specific αRep) and beads without biotinylated proteins (0) as a control. Beads were incubated with cell lysate containing EGFP and washed. Bound proteins were analysed by western blot using anti-GFP antibody followed by anti-rabbit horeseradish peroxydase (HRP) antibody. (**A**) Input: lysate before incubation with beads; Depleted cell lysate: lysate after beads incubation for each αRep and control. (**B**) αRep pull-down: analysis of proteins attached on beads after the washing step for the different samples.

#### αReps can be expressed in eukaryotic cells

αReps were initially developed using prokaryotic cells expression, but were not previously tested for expression in mammalian cells. In order to detect αRep expression in eukaryotic cells, the sequences coding for each bGFP and a non-relevant αRep were fused to the sequence coding for the fluorescent protein mCherry. The expression of the resulting αRep–mCherry fusion proteins was detected by fluorescence in transiently transfected live HEK cells. Red fluorescence could be seen in transfected cells for the four tested proteins, equally distributed in the cell cytoplasm (Figure 7A). No aggregate or cell toxicity due to the expression of αRep–mCherry could be observed during the experiment. The four αRep–mCherry fusions are expressed and stable in cell cytoplasm, no specific localization was apparent for those proteins, which appeared as freely diffusing in the whole cell.

**Figure 7:**
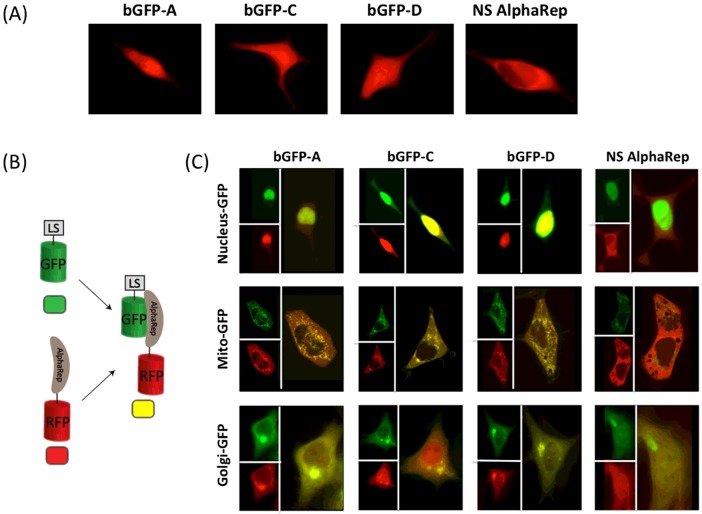
αReps characterizations in mammalian living cells (**A**) Plasmids expressing αRep–mCherry protein fusion (bGFP-A, bGFP-C, bGFP-D and NS αRep: non GFP-specific αRep) were transiently transfected in HEK cells. Protein expression was observed 24 h after transfection by confocal microscopy. The red fluorescence of the mCherry–αRep protein fusions is equally distributed inside the cell cytoplasm. (**B**) Scheme of co-localization experiment; LS: localizing sequence to nucleus, mitochondria and Golgi apparatus, fused to EGFP protein and observed in green in the cell; αRep–RFP: protein fusion of αRep–mCherry observed in red inside the cell. Co-localization of both fusion proteins resulting of the recognition of the GFP by the selected αRep can be observed by a yellow colour inside the cell. (**C**) Plasmids expressing αRep–mCherry and LS–GFP protein fusion were transiently co-transfected in mammalian cells. Each line shows representative cells expressing EGFP-fusion addressed to different subcellular compartment. GFP proteins were addressed into the nucleus (nucleus-GFP in HEK cells), the mitochondria (mito-GFP in HEK cells) and the Golgi apparatus (Golgi apparatus in HELA cells). Each column shows representative cells expressing an αRep–mCherry fusion. For each co-transfection, the same cell is imaged using (a) green fluorescence (EGFP), (b) red fluorescence αRep–mCherry and (m) merge of both images.

#### αReps recognize their target inside cells

The ability of αReps to discriminate their target within the context of a eukaryotic cell was then investigated. The three bGFPs and a non-relevant αRep control served as model proteins to compare the specific binding of αReps in different subcellular compartments. We used three different previously well characterized GFP fusions targeted to different cell compartments: NLS-GFP which is addressed to the nucleus [41], rab6A-GFP targeted to the Golgi apparatus [2] and Mito-GFP which is inserted into the outer membrane of the mitochondria [40]. HEK cells were co-transfected with one plasmid expressing localized EGFP and another expressing αRep–mCherry. Living cells were then observed by confocal microscopy following the EGFP and mCherry fluorescent proteins. Representative images of co-transfected cells are shown (Figures 7B and 7C) with red (αRep–mCherry) and green fluorescence (GFP) together with a merged image of the same cells to directly compare the localization of proteins. In each case, m-Cherry fused αRep binders are clearly co-localized with the differently addressed GFP fusions. The non-relevant αRep, as expected, showed no specific localization and was homogeneously distributed in the cytoplasm in presence of GFP fusion proteins. These results show that αReps are expressed, correctly folded and bind specifically their target in eukaryotic cells. The co-localization with nuclear GFP shows that the αRep proteins can also be targeted to the nucleus and that the nuclear target–αRep binder interaction is sufficient for nuclear localization, as αRep are not fused to a nuclear localization signal. Independently of their size and composition, the three bGFPs presented the same properties of stability and recognition of the GFP fusion proteins. αRep proteins therefore seem to be ideally suited to serve as specific binders in a eukaryotic cellular context.

## DISCUSSION

### GFP binders selection

The αRep library contains a repertoire of artificial repeat proteins from which specific binding molecules can be selected [26]. Using GFP as a model target protein, we further investigated the potential of the αRep library as a source of binders and their applicability as tools for protein target recognition in eukaryotic cells.

First, these results are an additional demonstration of the utility of scaffolds derived libraries: EGFP-specific αReps with different sequences and lengths were selected from a single phage display library. Detailed *in vitro* characterization confirmed that these binders tightly interact with EGFP. It is noteworthy that, although the sequence space created by the αRep randomization scheme is very large and far from being totally explored in the experimental library, the selections did produce a number of distinct interacting proteins with a useful affinity for practical applications. The GFP binders described here have been selected out from a single generic library, without any post-selection affinity maturation procedures. We therefore demonstrated that selection from a highly diverse ‘naive’ library could be efficient even without any previous immunization step. The target protein EGFP does not present any specific features that could favour specific interaction with αReps. Thus, the favourable properties of the described binders essentially result from the high chemical diversity of interaction surfaces embedded in the αRep library.

### Structure of EGFP–αRep complexes

Structural studies are essential to understand how scaffolds-derived binders precisely interact with their cognate target [31,46]. This was fully illustrated by the results of our crystallographic analysis of two bGFP–EGFP complexes.

The crystallization behaviour of the bGFP-A–EGFP and bGFP-C–EGFP complexes was radically different. Many buffer/precipitant conditions yielded bGFP-A–EGFP crystals formation whereas only one was found for bGFP-C–EGFP. Moreover, crystals appeared at different time scales: from a few hours for bGFP-A–EGFP up to many days for bGFP-C–EGFP. The structural differences between the two complexes and the size difference of the αReps critically influences their crystallization behaviour; bGFP-A contains three more internal repeats, producing an extended surface surrounding the EGFP molecule and therefore modifying the intermolecular contacts opportunities during crystal growth [47].

The αRep library was assembled by concatenation of microgenes leading to a variable number of protein repeats, which makes it unique among the documented artificial protein libraries. αRep proteins tend to make good quality protein crystals on their own but we have also shown that they can be used as crystallization chaperones for proteins that refuse to crystallize. The structure of fibronectin-binding protein E (FNE), a protein involved in S*treptococcus* pathogenic species could only be crystallized in complex with a specific αRep [31]. The fact that good quality crystals were obtained of complexes of the same target protein, with αRep proteins with a different number of repeats illustrates that the variable size of the proteins can be an asset.

The comparison of the structures of GFP in complex with bGFP-A and bGFP-C is very instructive. Using the same protein fold containing randomized residues at the same positions, αRep proteins were able to form very different complexes with the same target protein. The GFP surfaces bound by the two αRep proteins only partially overlap. The extended concave surface of bGFP-A is sufficiently large to accommodate the cylindrical shape of EGFP with an unexpected orientation, interacting with one end of the cylinder as well as with side chains located along the side of the barrel.

The structures of both αRep–GFP complexes clearly indicate that the target recognition is specific of the conformation of the native protein. This was also generally observed with binders selected out from other repeat protein libraries [17,19,26,31]. The variegated surface of αReps is located on the juxtaposed helices 2 on the concave side of the fold. The overall shape of this binding surface appears well adapted to bind large patches on folded proteins. The αRep scaffold is probably less well adapted to interact with clefts, crevices or any other type of concave features of protein targets.

The available structures of GFP/nanobodies [11,48] can be compared with GFP–αRep complexes. The EGFP surface interacting with bGFP-C fully overlap the surface of EGFP binding a Nanobody called ‘minimizer’, although the αRep binding surface is more extended. The same surface is therefore targeted with binders of different topologies. Although this could suggest that this part of EGFP has some intrinsic features, such as two exposed tyrosyl side chains, prone to be selected as anchoring residues, this is not decisive as others part of the EGFP surface are also efficiently targeted with VHH [11,49] as well as with αRep, as observed with bGFP-A.

The superposition of all known αRep structures [26,31] shows very little variations suggesting that the αRep fold is relatively rigid. This is in contrast with several natural HEAT repeat proteins that appear more flexible and adapt to their bound partners by local distortion of one repeat or by adjustments of inter-repeats contacts [50,51]. The limited flexibility of αReps, relatively to natural HEAT repeat proteins, is probably due to their high stability. The sequence definition procedure of consensus-based artificial repeat proteins like αRep (or DARPins) implicitly optimizes the protein stability, which in turn minimizes flexibility. However, the limited flexibility of these artificial folds does not appear to be an obstacle to the selection of high-affinity binders from these libraries. It seems however likely that additional affinity maturations step could be later used to further improve affinity of initially selected binders by optimization of the side chains compositions of the binding surface as well as the overall fold flexibility.

### Binders applications

These αRep-based GFP binders can be easily produced in large quantities and are very stable proteins. We show here that the affinity and specificity of three different αReps for their GFP target are sufficient to use them as new tools to purify GFP-fusion proteins from a cell extract in pull-down experiments.

Natural HEAT repeat proteins are found in various organisms from prokaryotes to humans. The restricted sub-family used for the consensus design of αRep is however more common in prokaryotic species. It was thus unclear how those artificial proteins would behave in a eukaryotic expression system. The intracellular eukaryotic expression of αRep variants confirms that they can be stably expressed in mammalian cells. No specific localization could be observed for αRep proteins. They are soluble in the cell cytoplasm and can diffuse into the nucleus.

All three αRep variants selected against the EGFP were able to co-localize with EGFP inside different cell compartments. Thus, αReps accurately report the localization of a target protein in living cells without forming any aggregates or causing toxicity, which are often observed when using intrabodies. Therefore, αRep proteins seem to be fully appropriate to explore intracellular processes in living cells, by interacting directly with endogenous proteins. As overexpression of recombinant protein binders inside the cell may induce a high background signal due to the presence of an excess of unbound binders. Strategies have been devised to match the expression level of the binder relatively to its endogenous target [16]. Here, cell lines were co-transfected with αRep and target constructs. When transfection is not applicable, alternative approaches such as viral delivery systems, cell penetrating peptides or ‘protein transfection systems’ using lipid-based delivery reagents could be used [9,52].

Detailed structural information provided by the crystal structures will provide the basis for future, more elaborate design. For example, the EGFP-binding αRep described in the present study could be easily fused to other targeted components to create hetero-bifunctional intracellular reagents. In this respect, the high foldability (low propensity to aggregation) of these αRep binders is critical both for efficient production and for their future use as generic EGFP-binding domains in engineered multi-domain proteins.

## Abbreviations

AR: adrenergic receptor
BCR-ABL: break point cluster region-gène abelson
DARPins: designed ankyrin repeat proteins
DMEM: Dulbecco's Modified Eagle medium
FNE: fibronectin-binding protein E
FP: fluorescent protein
GPCR: G-protein-coupled receptors
HEAT: Huntingtin, elongation factor 3 (EF3), protein phosphatase 2A (PP2A), and the yeast kinase (TOR1)
HEK: human embryonic kidney cells
HRP: horseradish peroxidase
IMAC: immobilized metal affinity chromatography
ITC: isothermal titration calorimetry
LRR: leucine rich-repeat
MCS: multiple cloning site
Ni-NTA: nickel-nitrilotriacetic acid
NLS: nuclear localization sequence
Rab6: ras-related in brain 6
ScFvs: single-chain variable fragment
SEC: size-exclusion chromatography
SH2: Src homology 2
SHP2: SH2 domain-containing phosphatase 2
SPR: surface plasmon resonance
TBST: Tris-buffered saline and Tween 20
TPR: tetratricopeptide
VHH: variable domain of heavy chain antibody
WB: western blot
